# Supplementary material for: Diabetes Prevalence and Associated Risk Factors among Women in a Rural District of Nepal Using HbA1c as a Diagnostic Tool: A Population-Based Study
Source: Int J Environ Res Public Health. 2022 Jun 8;19(12):7011. doi: 10.3390/ijerph19127011 (PMC9223207; doi:10.3390/ijerph19127011)
Supplement: Supplementary file 1 [file ijerph-19-07011-s001.zip › ijerph-1705067-supplementary.pdf]

S1 Table. Characteristics of women with and without HbA1c measurements

Characteristics		HbA1c measured	
	Total	Yes	No
	n (%) or mean ± SD		
**Total**	1498 (100.0)	757 (50.5)	741 (49.5)
**Age groups (years)**			
17-35	429 (28.6)	210 (27.7)	219 (29.6)
35-44	441 (29.4)	230 (30.4)	211 (28.5)
45-54	321 (21.4)	173 (22.9)	148 (20.0)
≥ 55	307 (20.5)	144 (19.0)	163 (22.0)
**Age (years)**	43.1 ± 14.3	43.0 ± 14.0	43.3 ± 14.7
**Ethnicity**			
Dalit	262 (17.5)	79 (10.5)	183 (24.7)
Adhivasi/Janajati	1056 (70.5)	596 (78.7)	460 (62.1)
Bramin/Chhetri	180 (12.0)	82 (10.8)	98 (13.2)
**Education**			
Uneducated	1280 (85.4)	663 (87.6)	617 (83.3)
Primary	94 (6.3)	44 (5.8)	50 (6.7)
Secondary	70 (4.7)	26 (3.4)	44 (5.9)
Higher	54 (3.6)	24 (3.2)	30 (4.0)
**Monthly household income (NPR)**	**n=1396**	**n=730**	**n=666**
≤ 24000	570 (40.8)	223 (30.5)	347 (40.8)
> 24000	826 (59.2)	507 (69.5)	319 (47.9)
**Number of children**	3.5 ± 1.9	3.5 ± 1.9	3.5 ± 1.8
**Parity**			
0	51 (3.4)	21 (2.8)	30 (4.0)
1	156 (10.4)	71 (9.4)	85 (11.5)
2	264 (17.6)	136 (18.0)	128 (17.3)
≥ 3	1027 (68.6)	529 (69.9)	498 (67.2)
**Family history of non-communicable diseases**			
Diabetes	25 (1.7)	11 (1.5)	14 (1.9)
CVDs/Hypertension	47 (3.2)	24 (3.2)	23 (3.2)
**Dietary factors**			
Vegetarian diet	115 (7.7)	35 (4.6)	80 (11.0)
Instant noodle intake	917 (61.7)	458 (60.6)	459 (62.9)
Instant noodle intake ≥ 2 times a week	332 (22.1)	251 (33.2)	81 (10.9)
Milk intake	918 (61.8)	451 (60.0)	467 (64.0)
Milk intake ≥ 2 times a week	440 (29.3)	289 (38.2)	151 (20.3)
**Smoking**			
Current	374 (25.0)	198 (26.2)	176 (23.8)
Former	99 (6.6)	46 (6.0)	53 (7.3)
Never	1022 (68.4)	513 (67.8)	509 (69.0)
**Anthropometric measurements**	**n=1395**	**n=717**	**n=678**
Height (cm)	149.0 ± 7.0	149.3 ± 6.9	148.8 ± 7.1
Weight (kg)	51.2 ± 10.1	50.7 ± 9.5	51.7 ± 10.5
Body mass index (kg/m^2)	23.0 ± 4.7	22.8 ± 4.4	23.4 ± 5.1
Waist circumference (cm)	77.1 ± 8.9	77.8 ± 9.1	76.4 ± 8.6
